# KCP10043F Represses the Proliferation of Human Non-Small Cell Lung Cancer Cells by Caspase-Mediated Apoptosis via STAT3 Inactivation

**DOI:** 10.3390/jcm9030704

**Published:** 2020-03-05

**Authors:** Jeong-Hun Lee, Hwi-Ho Lee, Ki Deok Ryu, Misong Kim, Dohyeong Ko, Kyung-Sook Chung, Ahmed H.E. Hassan, Seung Hyeun Lee, Jae Yeol Lee, Kyung-Tae Lee

**Affiliations:** 1 Department of Pharmaceutical Biochemistry, College of Pharmacy, Kyung Hee University, Seoul 02447, Korea; ztztzt08@hanmail.net (J.-H.L.); hhlee4083@naver.com (H.-H.L.); adella76@hanmail.net (K.-S.C.); 2 Department of Life and Nanopharmaceutical Sciences, Graduate School, Kyung Hee University, Seoul 02447, Korea; 3 BioCast R&D Center, 702, Eonju-ro, Gangnam-gu, Seoul 06061, Korea; 4 Research Institute for Basic Sciences and Department of Chemistry, College of Sciences, Kyung Hee University, Seoul 02447, Korea; ryukd0201@gmail.com (K.D.R.); miisong@khu.ac.kr (M.K.); ajwl6502@khu.ac.kr (D.K.); 5 Department of Medicinal Chemistry, Faculty of Pharmacy, Mansoura University, Mansoura 35516, Egypt; ahmed_hassan@mans.edu.eg; 6 Division of Pulmonary and Critical Care Medicine, Department of Internal Medicine, Kyung Hee University Medical Center, Kyung Hee University School of Medicine, Seoul 02447, Korea; humanmd04@hanmail.net; 7 KHU-KIST Department of Converging Science and Technology, Kyung Hee University, Seoul 02447, Korea

**Keywords:** STAT3, apoptosis, NSCLC, caspase, mitochondrial membrane potential

## Abstract

We previously reported that 4-(4-fluorobenzylcarbamoylmethyl)-3-(4-cyclohexylphenyl)-2-[3-(*N,N*-dimethylureido)-*N′*-methylpropylamino]-3,4-dihydroquinazoline (KCP10043F) can induce G_1_-phase arrest and synergistic cell death in combination with etoposide in lung cancer cells. Here, we investigated the underlying mechanism by which KCP10043F induces cell death in non-small cell lung cancer (NSCLC). Propidium iodide (PI) and annexin V staining revealed that KCP10043F-induced cytotoxicity was caused by apoptosis. KCP10043F induced a series of intracellular events: (1) downregulation of Bcl-2 and Bcl-xL and upregulation of Bax and cleaved Bid; (2) loss of mitochondrial membrane potential; (3) increase of cytochrome *c* release; (4) cleavage of procaspase-8, procaspase-9, procaspase-3, and poly (ADP-ribose) polymerase (PARP). In addition, KCP10043F exhibited potent inhibitory effects on constitutive or interleukin-6 (IL-6)-induced signal transducer and activator of transcription (STAT3) phosphorylation and STAT3-regulated genes including survivin, Mcl-1, and cyclin D_1_. Furthermore, STAT3 overexpression attenuated KCP10043F-induced apoptosis and the cleavage of caspase-9, caspase-3, and PARP. Docking analysis disclosed that KCP10043F could bind to a pocket in the SH2 domain of STAT3 and prevent STAT3 phosphorylation. The oral administration of KCP10043F decreased tumor growth in an A549 xenograft mouse model, as associated with the reduced phosphorylated STAT3, survivin, Mcl-1, and Bcl-2 expression and increased TUNEL staining and PARP cleavage in tumor tissues. Collectively, our data suggest that KCP10043F suppresses NSCLC cell growth through apoptosis induction via STAT3 inactivation.

## 1. Introduction

Lung cancer is one of the most frequent cancers and the leading cause of worldwide cancer-related mortality. Non-small cell lung cancer (NSCLC) accounts for over 85% of lung cancer cases [1,2]. Despite the development of advanced techniques in surgery, radiotherapy, and chemotherapy, anomalies such as the mutations or amplification of epidermal growth factor receptor (EGFR), mutation of Kirsten rat sarcoma viral oncogene homolog (KRAS), and high expression levels of multidrug transporters remain a major problem in the treatment of NSCLC [3]. Therefore, there is an essential need for a safe, effective, and affordable therapeutics to treat this disease.

Apoptosis is an orchestrated and programmed cellular process, which plays a critical part in the cause and development of certain diseases [4]. Therefore, the induction of apoptosis is one of the strategies in cancer treatment such as irradiation or chemotherapy [5]. Two major signaling pathways induce apoptosis. One is an intrinsic pathway mediated by stimuli targeting the mitochondria, which induce mitochondrial outer membrane permeabilization regulated by B cell lymphoma (Bcl) family proteins such as Bcl-2, Bcl-xL, and other pro-apoptotic or anti-apoptotic proteins, while the other is an extrinsic pathway engaged with extracellular ligands such as TNF (tumor necrosis factor), Fas-L (Fas ligand), and TRAIL (TNF-related apoptosis-inducing ligand) which are attached to the extracellular domain of their specific receptors [6]. 

The signal transducer and activator of transcription (STAT) pathway is a molecular signaling pathway that communicates the biological effect of external stimuli such as cytokines and growth factors and controls physiological systems such as cell growth, differentiation, senescence, and apoptotic cell death [7]. STAT3 is mainly activated in several cancers including lung cancer [8,9]. Various receptor tyrosine kinases (RTKs) with intrinsic tyrosine kinase activity stimulate tyrosine phosphorylation of STAT3, including the human epidermal growth factor receptor (EGFR) family of receptors, vascular endothelial growth factor receptor (VEGFR), and Janus kinases (JAKs) [10]. Advanced studies have shown that STAT3 plays a vital role in restricting apoptotic cell death and promoting cell proliferation during tumor development [11]. Therefore, the suppression of the STAT3 signal transduction pathway has been investigated as a cancer therapeutic strategy. Anti-cancer drugs such as icotinib can inhibit lung cancer development mediated by the EGFR-JAK-STAT3 transduction pathway [12].

The T-type calcium channel is expressed on the plasma membrane of cells in diverse tissues [13] and plays a vital role in a variety of neurophysiological and pathological functions such as cardiovascular and nervous system functions [14]. However, recent studies have shown that the T-type calcium channel is related to the proliferation of tumor cell [15], and the expression of T-type calcium channel is correlated with cancer growth and progression [16]. Therefore, the inhibition or downregulation of the T-type calcium channel could lead to the suppression of cancer cell proliferation or the induction of cancer cell death [17,18,19,20,21].

We previously reported that 4-(4-fluorobenzylcarbamoylmethyl)-3-(4-cyclohexylphenyl)-2-[3-(*N,N*-dimethylureido)-*N′*-methylpropylamino]-3,4-dihydroquinazoline (KCP10043F) can induce cell cycle arrest by the downregulations of cyclin-dependent kinase (CDK) 2, CDK4, CDK6, cyclin D_2_, cyclin D_3_, and cyclin E at the protein levels and synergistic inducing apoptosis with etoposide in A549 human lung cancer cells [22]; however, anti-proliferative potential or the underlying mechanism of this compound has not been determined. Therefore, we studied the molecular mechanism underlying the anti-proliferative properties of KCP10043F by examining its effect on STAT3 in NSCLC cells and the mouse model bearing xenografts of A549 human lung cancer.

## 2. Materials and Methods

### 2.1. Chemical and Reagents

**Figure 1:**
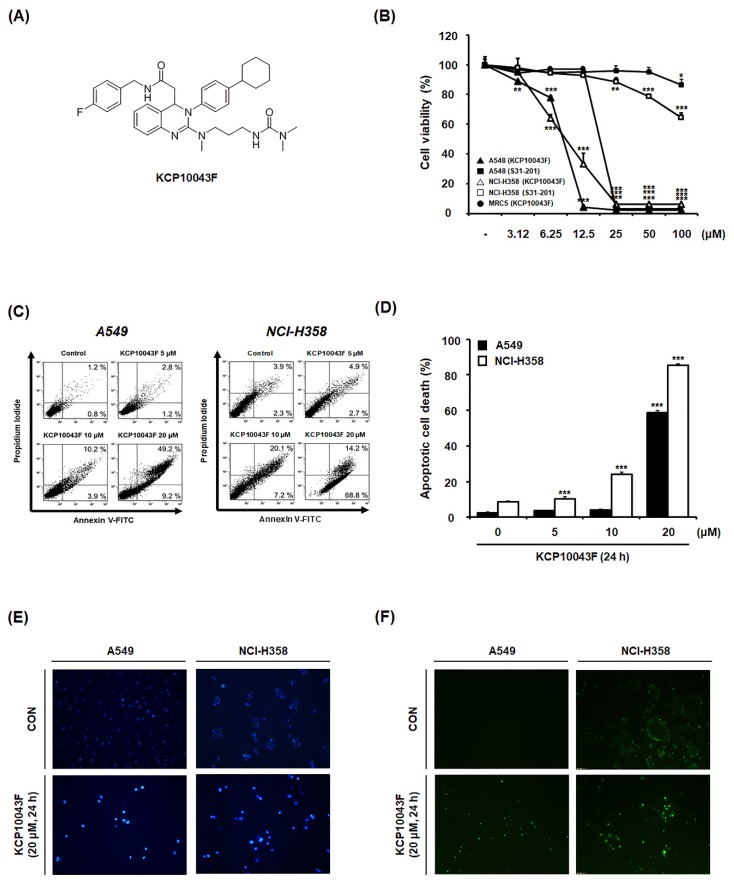
Induction of apoptosis by KCP10043F in A549 and NCI-H358 cells. (**A**) Structure of KCP10043F. (**B**) A549, NCI-H358, and MRC5 cells were treated with KCP10043F (3.12–100 μM) for 48 h. S3I-201 (3.12–100 µM) was used as a positive control with A549 and NCI-H358 cells. (**C)** A549 and NCI-H358 cells were treated with KCP10043F (5, 10, or 20 µM) for 24 h and co-stained with propidium iodide (PI) and fluorescein isothiocyanate (FITC)-conjugated annexin V for detecting apoptosis by flow cytometry. (**D**) The portion of early apoptosis (Annexin^+^/PI^−^) cells and late apoptosis (Annexin^+^/PI^+^) cells in the graph is determined as apoptotic cell death rate. (**E,F**) A549 and NCI-H358 cells were treated with 20 μM KCP10043F for 24 h. DNA fragmentation was detected by DAPI and TUNEL assay. Data represent the mean ± standard deviation (SD) of the results from three independent experiments. ** *p* < 0.01, *** *p* < 0.001 vs. untreated control group.

KCP10043F (Figure 1A) used in this study was synthesized as previously reported [22]. Acrylamide and bis-acrylamide and ammonium persulfate were purchased from Bio-rad Laboratories (Hercules, CA, USA). Antibody against caspase-8 (551242) and FITC (fluorescein isothiocyanate)-Annexin V Apoptosis Detection Kit I were purchased from BD Bioscience pharmigen (San Jose, CA, USA). Carbonyl cyanide *m*-chlorophenyl hydrazine (CCCP), 4′,6-diamidino-2-phenylindole (DAPI), 3,3′-dihexyloxacarbocyanine iodide (DiOC_6_), phenylmethylsulfonyl fluoride (PMSF), propidium iodide (PI), *N,N,N′,N′*-tetramethylethylenediamine (TEMED), and ribonuclease A (RNase A) were purchased from Sigma Aldrich (St. Louis, MO, USA). Antibodies against α-tubulin (sc-5286), β-actin (sc-81178), Bax (sc-7480), Bcl-2 (sc-7382), Bcl-xL (sc-8392), caspase-3 (sc-7272), Mcl-1 (sc-69839), cyclin D_1_ (sc-8396), poly (ADP-ribose) polymerase (PARP) (sc-8007), survivin (sc-17779) and total STAT3 (sc-8019) were purchased from Santa Cruz Biotechnology Inc (Santa Cruz, CA, USA). Antibodies against Bid (#2002), caspase-9 (#9502), cleaved caspase-3 (#9661), COX-4 (#4844), cytochrome *c* (#11940), and phospho-STAT3 (Y705) (#9145) were purchased from Cell Signaling Technology (Danvers, MA, USA). Mounting Medium with DAPI was purchased from Vector Laboratories (Burlingame, CA, USA). Lipofectamine™ Transfection Reagent was obtained from Thermofisher Scientific (Waltham, MA, USA). z-VAD-fmk (z-Val-Ala-Asp-fluoromethylketone) was obtained from MP Biomedicals (Santa Ana, CA, USA). 

### 2.2. Cell Culture

A549 (human lung carcinoma cell), National Cancer Institute (NCI)-H358 (human bronchioalveolar carcinoma cell), and MRC5 (human lung fibroblast) were obtained from the Korean Cell Line Bank (Seoul, Korea). A549 and NCI-H358 cells were cultured in Rosewell Park Memorial Institute (RPMI) 1640 medium and MRC5 cells were cultured in minimum essential media (MEM) with 10% inactivated FBS (fetal bovine serum) and 1% penicillin (100 units/mL) and streptomycin sulfate (100 µg/mL). All cells were cultured under the condition of 5% CO_2_ at 37 °C.

### 2.3. Cytotoxicity Assay

The 3-(4,5-dimethylthiazolyl-2)-2,5-diphenyltetrazolium bromide (MTT) assay was used as previously described to examine cytotoxicity [23]. briefly, cells were seeded in a 96-well plate, and each well contains 5 × 10^4^ cells/mL in 100 µL of the medium. After incubation for 24 h, serial concentrations of KCP10043F were treated in triplicate. After treatment for 48 h, 20 µL MTT solution was consecutively treated and cells in the plate were incubated for a 4 h in the dark. The medium was removed and cell-forming formazan blue was dissolved with 200 µL of dimethyl sulfoxide (DMSO). Optical density was measured by enzyme-linked immunosorbent assay (ELISA) at 540 nm.

### 2.4. Annexin V-FITC (Fluorescein Isothiocyanate) and Propidium Iodide (PI) Double Staining Assay

To detect the induction of apoptosis, KCP10043F-treated or untreated cells were harvested by using trypsin and washed twice with phosphate-buffered saline (PBS). The pellets were re-suspended in 100 µL annexin V binding buffer with FITC–conjugated annexin V and PI solution and incubated for 15 min in dark. Then stained cells were analyzed by fluorescence-activated cell sorting (FACS) cytometer, Cytomics FC 500 (Beckman Coulter, CA, USA).

### 2.5. DAPI (4′,6-Diamidino-2-Phenylindole) Staining Assay

To observe DNA fragmentation, KCP10043F-treated cells were harvested and washed with PBS. After being fixed in 4% formaldehyde solution for 10 min and stained with DAPI for an additional 10 min, apoptotic cells were detected by Olympus IX51 fluorescent microscope (Olympus, Tokyo, Japan) through characteristics of apoptosis (e.g., nuclear condensation, the formation of membrane blebs and apoptotic bodies).

### 2.6. Terminal Deoxynucleotidyl Transferase dUTP Nick end Labeling (TUNEL) Assay

KCP10043F-treated cells underwent fixing and permeabilization process or tumor tissues were fixed 10% paraformaldehyde and embedded in paraffin and then reacted TUNEL mixture according to the manufacturer’s instruction (in situ cell death detection kit, POD, Roche, Germany). The stained slides were rinsed with PBS three times and mounted with mounting medium, detected by Olympus IX51 fluorescent microscope (Olympus, Tokyo, Japan).

### 2.7. Western Blot Analysis

To investigate the alteration of protein expression, KCP10043F-treated cells were collected and lysed in PRO-PREP^TM^ protein lysis buffer (Intron Biotechnology, Seongnam, Korea) for 30 min at 4 °C. The protein concentration was determined by Bradford assay reagent. Cell extract was fractionated by 8–15% sodium dodecyl sulfate–polyacrylamide gel electrophoresis (SDS–PAGE) and transferred onto polyvinylidene difluoride (PVDF) membrane, which incubated for 1 h in blocking solution at room temperature. The membrane was incubated in non-fat dry milk with the primary antibody at 4 °C overnight. Blots were washed three times with Tris-buffered saline (TBS) containing 0.1% Tween-20 and incubated with horseradish peroxidase (HRP)-conjugated secondary antibody for 2 h at room temperature, rewashed three times with Tween 20/Tris-buffered saline and detected using enhanced chemiluminescence detection system (Amersham, Buckinghamshire, England).

### 2.8. Mitochondrial and Cytosolic Fractionation

The mitochondrial and cytosolic fraction proceeded following the instruction in Mitochondria Isolation Kit for cultured cell (Thermofisher Scientific, MA, USA). Briefly, KCP10043F-treated cells were collected and homogenized by Dounce Glass Grinder (Kimble-Chase, NJ, USA) and additional processes isolated cytosol and mitochondrial fraction from homogenized cell lysis buffer.

### 2.9. Measurement of Mitochondrial Membrane Potential (ΔΨ_m_)

To measure the changes of *ΔΨm* in KCP10043F-treated cells, the cells were stained with 40 nM DiOC_6_ for 30 min at 37 °C in dark. The stained cells were collected and washed twice with PBS, then detected by a FACS cytometer.

### 2.10. Transfection for Signal Transducer and Activator of Transcription (STAT3) Overexpression

To enhance STAT3 expression, the cells were incubated in Opti-MEM media (Thermofisher Scientific, MA, USA) and supplemented the mixtures of pMXs-STAT3-C, pMXs-gw plasmid (Addgene, MA, USA) and Lipofectamine™ Transfection Reagent (Thermofisher Scientific, MA, USA). After transfection, cells were treated with KCP10043F and confirmed by Western blot for checking overexpression.

### 2.11. Cytokine Production

To quantify the production of cytokine, supernatants of KCP10043F-treated cells were collected and analyzed by ELISA kits (BD Bioscience, CA, USA) according to manufacturer instruction.

### 2.12. Immunocytochemistry 

After co-treatment with KCP10043F and interleukin-6 (IL-6), cells were washed with PBS and then were fixed with 4% paraformaldehyde in PBS at 4 °C. The next day, to induce cell permeability, cells were washed with PBS three times and were incubated with 0.3% Triton-X 100/PBS for 1 h at 25 °C as described previously [23]. counterstaining with DAPI. After glycerol mounting, images were captured using the K1-Fluo laser scanning confocal microscope (NANOSCOPE systems, Seoul, Republic of Korea).

### 2.13. Animals

The male Bagg Albino (BALB)/c nude mice (7-week-old, 20–23 g) were obtained from Nara Biotec Co. (Pyeongtaek, Republic of Korea). All animal experiments were approved by the Committee for the Care and Use of Laboratory Animals in the Kyung Hee University (KHUASP(SE)-18–146) and were performed according to the National Institutes of Health (NIH) and Kyung Hee University Guidelines for Laboratory Animals Care and Use as described previously [23]. The animal protocol was designed to minimize pain and discomfort to the animals. The animals were acclimatized to laboratory conditions for more than one week prior to experimentation.

### 2.14. In Vivo Tumor Xenograft Studies

To establish the A549 xenograft model, subcutaneous implantation of A549 cells was performed as previously described [23]. Tumor size was checked with a caliper three times per week and calculated as V = π/6 × (length) × (width)^2^ [24]. When tumor volume reached about 250 mm^3^, mice were divided into 5 groups (n = 7) and treated with (Group 1); vehicle (Ethanol:Cremophor:D.W. = 1:1:18, per oral (p.o)), (Group 2); paclitaxel (positive control, 5 mg/kg, intraperitoneal (i.p.)) and (Group 3–5); KCP10043F (5, 15, or 30 mg/kg daily, p.o., respectively). During the treatment, tumor volume and body weight were measured three times per week. On day 27, mice were killed and tumors were obtained.

### 2.15. Molecular Docking Analysis

Compound KCP10043F (S and R stereoisomers) was sketched, hydrogens added, energy minimized and saved as mol2 files. SwissDock which is based on the docking software EADock DSS running on the Vital-IT cluster was used to dock blindly KCP10043F over the whole STAT3 crystal structures (Protein Data Bank code: 3cwg) in accurate mode [25,26,27]. The retrieved docked poses were visually analyzed and assessed.

### 2.16. Statistical Analysis

Data are represented as the mean ± SD. of triplicate experiments. Statistical significances were identified using analysis of variance (ANOVA) and Dunnett’s post hoc test, and *p*-values of less than 0.05 were reputed statistically significant.

## 3. Results

### 3.1. KCP10043F Inhibits the Proliferation of A549 and NCI-H358 Human Non-Small Cell Lung Cancer (NSCLC) Cells by Inducing Apoptosis

We previously reported that KCP10043F attenuated G_1_ cell cycle progression and caused apoptotic cell death when co-treated with etoposide in A549 cells [22]. To determine the concentration of KCP10043F that causes cell death, we treated A549 and NCI-H358 cells with various concentrations (3.15–100 µM) of KCP10043F for 48 h. As shown in Figure 1B, the IC_50_ values of KCP10043F against A549 and NCI-H358 cells (8.23 ± 0.34 µM and 9.23 ± 0.53 µM, respectively) were lower than the IC_50_ values of KCP10043F against MRC5 normal human lung fibroblast cells (18.89 ± 0.68 µM) and S3I-201 (STAT3 inhibitor) [28] against A549 cells (192.67 ± 4.38 µM) and NCI-H358 cells (149.63 ± 2.75 µM). Next, to investigate whether the cytotoxicity of KCP10043F is related to the generation of apoptosis, we examined the externalization of phosphatidylserine (PS) by PI and annexin V double staining and assessed DNA fragmentation by DAPI and TUNEL assay. The results showed that KCP10043F markedly increased the percentage of cells in early apoptosis (Annexin^+^/PI^−^) cells and late apoptosis (Annexin^+^/PI^+^) cells in a concentration- and time-dependent manner (Figure 1C,D, and Figure S1). Furthermore, DAPI and TUNEL staining revealed that KCP10043F caused DNA fragmentation (Figure 1E,F), suggesting that KCP10043F induce human NSCLC cell death via apoptosis rather than non-specific necrosis.

### 3.2. KCP10043F Induces Caspase-Dependent Apoptosis 

**Figure 2:**
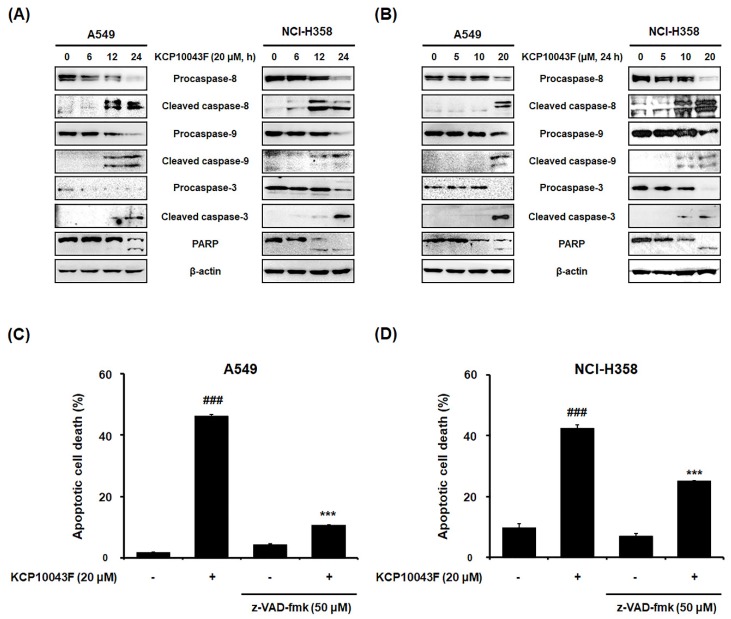
Activation of the caspase-dependent pathway by KCP10043F in A549 and NCI-H358 cells. (**A**) Cells were treated with 20 µM KCP10043F for the indicated times (6, 12, or 24 h) and (**B**) treated with the indicated concentrations (5, 10, or 20 µM) of KCP10043F for 24 h. The cells were harvested, and total cell lysates were prepared. The expression levels of procaspase-8, cleaved caspase-8, procaspase-9, cleaved caspase-9, procaspase-3, cleaved caspase-3, and poly (ADP-ribose) polymerase (PARP) were examined by Western blot analysis. β-actin was used as an internal control. (**C**,**D**) A549 and NCI-H358 cells with pretreated with a broad-caspase inhibitor (z-Val-Ala-Asp-fluoromethylketone (z-VAD-fmk)) for 1 h, followed by treatment with 20 μM KCP10043F for 24 h. The cells were co-stained with annexin V-FITC and PI, and apoptosis was detected by flow cytometry. Data represent the mean ± SD of the results from three independent experiments. ^###^ *p* < 0.001 vs. untreated control group, *** *p* < 0.001 vs. KCP10043F-treated group.

As apoptosis occurs through the intrinsic and/or extrinsic pathways [29], we examined the cleavage of procaspases and PARP by Western blot analysis. After treatment with 20 µM KCP10043F for 24 h, not only the active forms of caspase-8 and caspase-9 but also the cleaved forms of caspase-3 and PARP were increased (Figure 2A,B), suggesting the activation of both the extrinsic and intrinsic apoptotic pathways. Next, to determine the involvement of the caspase-dependent pathway, pretreatment with 50 µM z-VAD-fmk (broad caspase inhibitor) significantly attenuated KCP10043F-induced apoptosis in both lung cancer cell lines (Figure 2C,D). These results suggest that the caspases play an essential role in KCP10043F-induced apoptosis in A549 and NCI-H358 cells.

### 3.3. KCP10043F Induces the Release of Cytochrome C into the Cytosol and Loss of ΔΨ_m_

**Figure 3:**
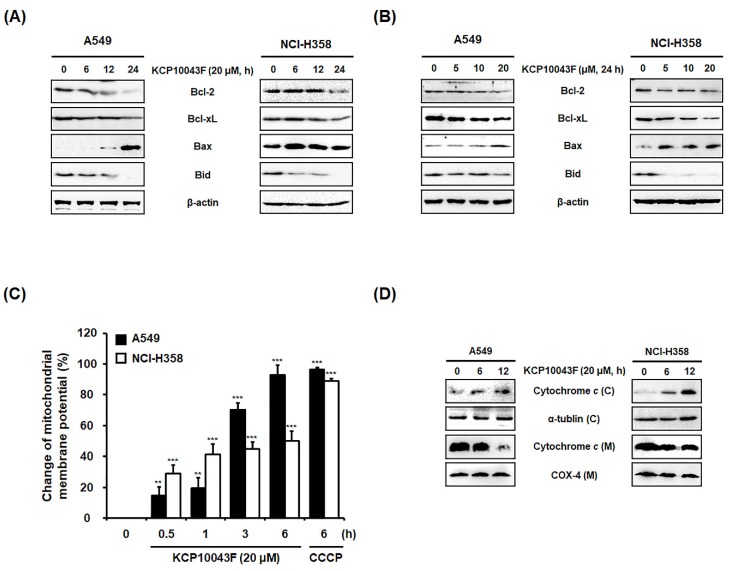
Regulation of mitochondria-related proteins by KCP10043F in A549 and NCI-H358 cells. (**A**) Cells were treated with 20 µM KCP10043F for the indicated times (6, 12, or 24 h) and (B) treated with KCP10043F (5, 10, or 20 µM) for 24 h. The cells were harvested, and total cell lysates were prepared. Total cellular proteins were prepared, resolved by sodium dodecyl sulfate–polyacrylamide gel electrophoresis (SDS–PAGE), and detected using specific Bcl-2, Bcl-xL, Bax, and Bid antibodies. β-actin was used as an internal control. (**C**) After treatment with 20 μM KCP10043F for the indicated times (0.5, 1, 3, or 6 h), cells were stained with DiOC_6_ (40 nM) for 30 min and detected by flow cytometry. Carbonyl cyanide *m*-chlorophenyl hydrazine (CCCP, 50 µM) was used as a positive control. Data represent the mean ± SD of the results from three independent experiments. ** *p* < 0.01, *** *p* < 0.001 vs. untreated control group. (**D**) Cells were treated with 20 µM KCP10043F for the indicated times (6 or 12 h). Mitochondrial (M) and cytosolic (**C**) fractions were prepared as described in the *Materials and Methods* Section. Cytosolic and mitochondrial proteins were prepared, resolved by SDS–PAGE, and detected using specific cytochrome *c* antibodies. COX-4 and α-tubulin were used as internal controls.

Considering the important players of the mitochondrial pathway in apoptosis, we examined changes in the members of Bcl-2 family proteins in KCP10043F-treated A549 and NCI-H358 cells. As shown in Figure 3A,B, KCP10043F reduced the expression of the Bcl-2 and Bcl-xL, and increased the expression of the pro-apoptotic proteins, Bax and cleaved Bid, concurrently. It is known that the changes in the expression of Bcl-2 family members induce the dissipation of *ΔΨm* and the release of mitochondrial pro-apoptotic proteins. Following treatment with KCP10043F (20 μM), the loss of *ΔΨ_m_* was time-dependently increased (Figure 3C). Similar to KCP10043F, CCCP which is widely used uncoupler of mitochondrial oxidative phosphorylation, also induced *ΔΨm* depolarization. Furthermore, the expression levels of cytosolic cytochrome *c* were significantly increased after KCP10043F treatment (Figure 3D). These results suggest that KCP10043F induces the loss of *ΔΨm* and the translocation of mitochondrial cytochrome *c* to the cytosol by unbalancing Bcl-2 family protein levels, which resulted in the activation of caspases-9 and -3.

### 3.4. KCP10043F Affects the STAT3 Signaling Pathway in A549 and NCI-H358 Cells

**Figure 4:**
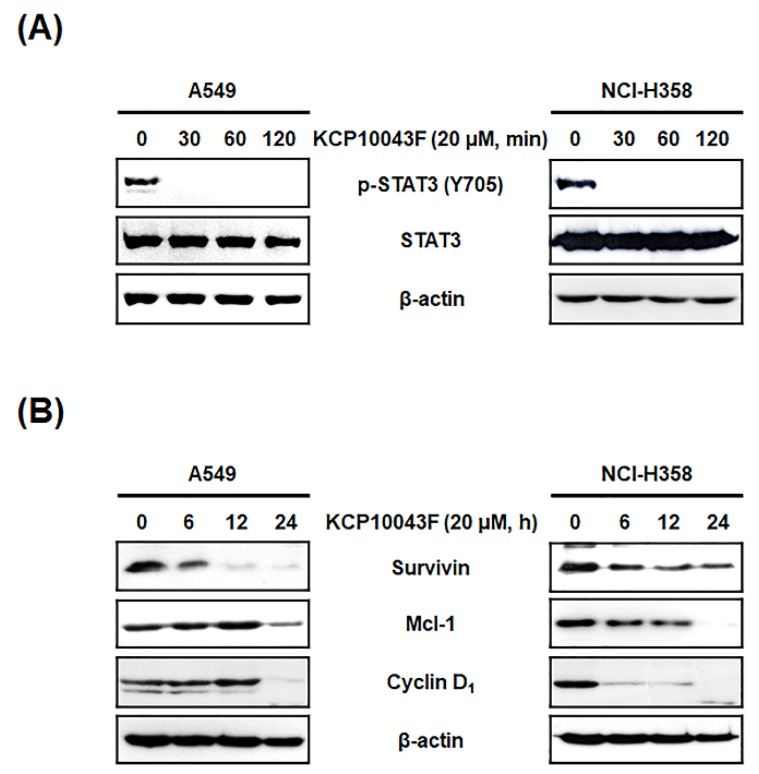
Suppression of the signal transducer and activator of transcription (STAT3) signaling pathway by KCP10043F in A549 and NCI-H358 cells. (**A**) After treatment with 20 μM KCP10043F for the indicated times (30, 60, or 120 min), total cell lysates were prepared and analyzed using specific p-STAT3 and STAT3 antibodies. (**B**) Cells were treated with 20 µM KCP10043F for the indicated times (6, 12, or 24 h). Total cellular proteins were prepared, resolved by SDS–PAGE, and detected using specific survivin, Mcl-1, and cyclin D_1_ antibodies. β-actin was used as an internal control.

Constitutive STAT3 activation has been observed in several lung cancer cells and tissues [9], and the restriction of STAT3 activation in a tumor can suppress the expression of pro-proliferative, angiogenetic, and anti-apoptotic genes [30]. To investigate whether KCP10043F-induced apoptosis is associated with the inhibition of constitutive STAT3 activation, we examined the phosphorylation of STAT3 (Y705) by Western blotting. Notably, KCP10043F reduced the levels of phosphorylated STAT3 (p-STAT3) with no changes in the expression levels of total STAT3 (Figure 4A). STAT3 is known to transcriptionally regulate apoptosis-related proteins such as survivin, Mcl-1, and cyclin D_1_ [31,32]; as expected, KCP10043F decreased the expression levels of these proteins in A549 and NCI-H358 cells (Figure 4B and Figure S2). 

**Figure 5:**
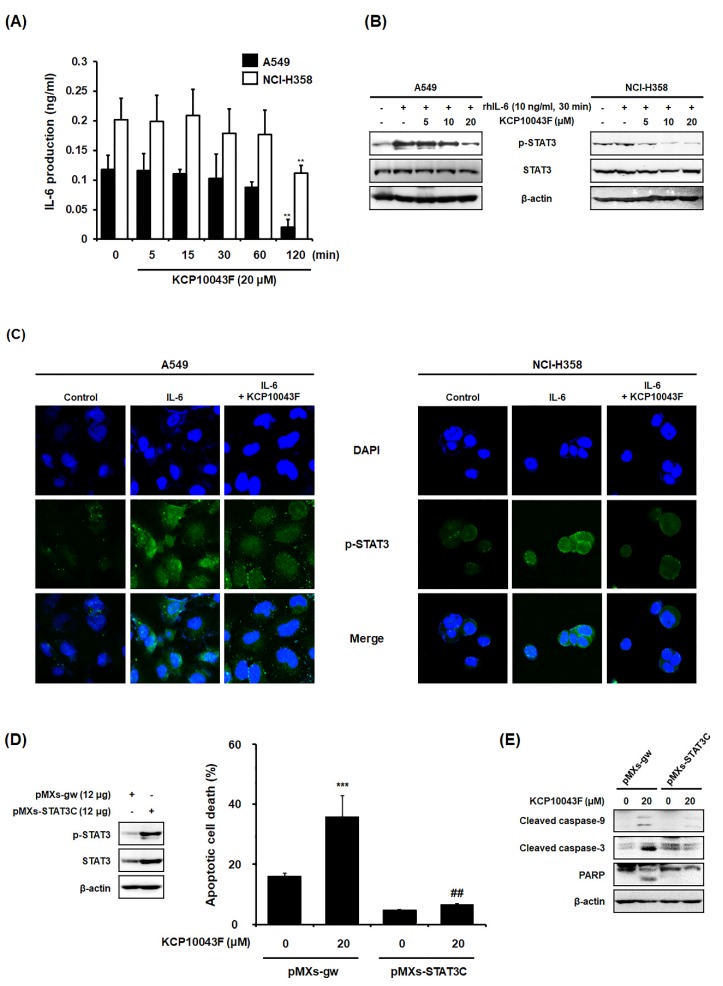
Effect of KCP10043F on the IL-6-induced STAT3 signaling pathway in A549 and NCI-H358 cells. (**A**) A549 and NCI-H358 cells were treated with 20 μM KCP10043F for the indicated times (5, 15, 30, 60, or 120 min). IL-6 production in the cell culture media was quantified using an EIA kit. Data represent the mean ± S.D. of the results from three independent experiments. ** *p* < 0.01 vs. untreated control group. After pretreatment with KCP10043F (5, 10, or 20 μM) for 1 h, the cells were stimulated with recombinant human IL-6 (10 ng/mL) for 30 min, and STAT3 activation was determined by (**B**) Western blot and (**C**) immunofluorescence analyses. Cells were transfected with pMXs-gw and pMXs-STAT3C for the overexpression of STAT3. (**D**) Transfected A549 cells were treated with 20 μM KCP10043F for 12 h and analyzed by flow cytometry with annexin V-FITC and PI staining. Data represent the mean ± SD. of the results from three independent experiments. *** *p* < 0.001 vs. pMXs-gw-transfected control group. ^##^ *p* < 0.001 vs. KCP10043F-treated control group. (**E**) Western blot analyses were performed to detect apoptotic cell death.

Furthermore, the production of the cytokine IL-6 (an inducer of STAT3) was quantified, which showed a decreasing trend in a time-dependent manner (Figure 5A). Consistent with the above results, the inhibitory effect of KCP10043F on the phosphorylation of STAT3 was also observed in cells stimulated with IL-6 as demonstrated by the Western blotting and the green fluorescence (IL-6-induced STAT3 phosphorylation) (Figure 5B,C). To confirm whether the pro-apoptotic effects of KCP10043F are associated with STAT3, we evaluated the apoptotic effects of KCP10043F on STAT3-overexpressed A549 human lung cancer cells with pMXs-STAT3C transfection. STAT3 was ectopically overexpressed by transfection, and overexpressed STAT3 and p-STAT3 levels in A549 cells were analyzed by Western blot analysis (Figure 5D). As shown in Figure 5D and E, KCP10043F-induced apoptotic cell death, and cleaved caspase-9, caspase-3, and PARP were reduced in STAT3-overexpressed cells compared with KCP10043F-treated control cells. Collectively, these results suggest that KCP10043F could specifically suppress p-STAT3 and STAT3-regulated anti-apoptotic proteins including survivin, Mcl-1, and cyclin D_1_, contributing to the induction of apoptosis.

### 3.5. KCP10043F Docks into the SH2 Domain of STAT3

**Figure 6:**
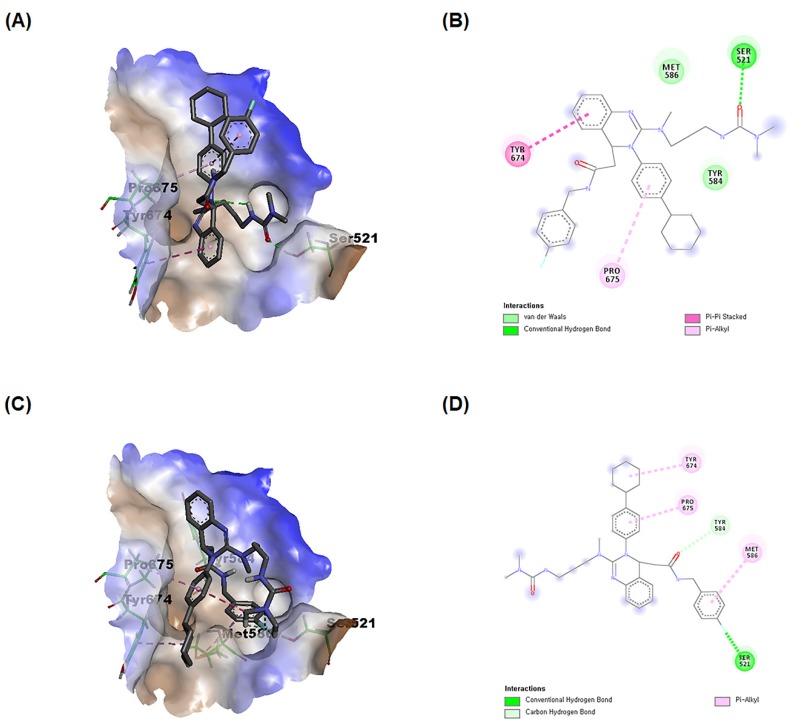
Interaction of KCP10043F with the SH2 domain of STAT3. (**A**) Docking of the S stereoisomer of KCP10043F into the SH2 domain of 3cwg. (**B**) Interactions of the S stereoisomer of KCP10043F within the binding pocket of 3cwg. (**C**) Docking of the *R* stereoisomer of KCP10043F into the SH2 domain of 3cwg. (**D**) Interactions of the *R* stereoisomer of KCP10043F within the binding pocket of 3cwg.

The STAT3 core structure consists of six domains: N-domain (ND), coiled-coil domain (CCD), DNA-binding domain (DBD), linker domain, SH2 domain, and transcriptional activation domain [33]. The literature has revealed that STAT3 inhibitors could be SH2 domain inhibitors or DBD inhibitors [34]. To characterize the type of STAT3 inhibition by KCP10043F, a blind docking study was performed with the whole crystal structure of STAT3 to determine potential binding sites. The results of the docking study showed that both the S and R KCP10043F stereoisomers could fit exclusively within the SH2 domain (Figure 6A–D). The calculated binding free energies and KCP10043F-STAT3 complex energy for the S stereoisomer were more favorable compared with those for the R stereoisomer, indicating that the S stereoisomer of KCP10043F contained more potential STAT3 SH2 domain binders (binding energy ΔG = −7.79 kcal/mol and ligand-protein complex energy of −3230.37 kcal/mol for the S stereoisomer vs. binding energy ΔG = −7.39 kcal/mol and ligand-protein complex energy of −3227.35 kcal/mol for the R stereoisomer). These results showed that the KCP10043F could bind to a pocket in the SH2 domain of STAT3; thus, it might function as an SH2 domain of STAT3 inhibitor.

### 3.6. KCP10043F Suppresses Tumor Growth through Apoptotic Cell Death in A549 Xenograft Model

**Figure 7:**
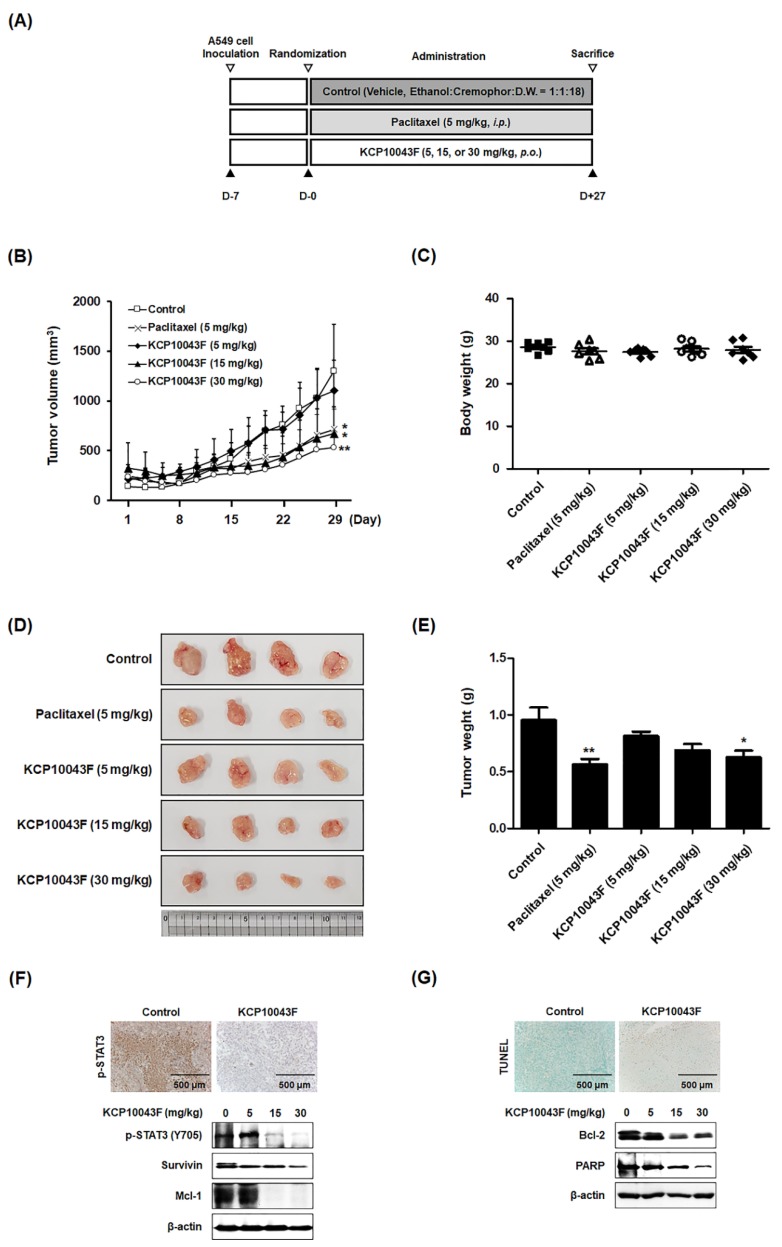
Antitumor effects of KCP10043F in human lung cancer xenograft mouse model by the induction of apoptosis. (**A**) Experimental design of the in vivo A549 xenograft model. A549 cells were subcutaneously inoculated into 7-week-old BALB/c nude mice (*n* = 7 per group), and the mice were treated orally with the vehicle (every day for 5 days), paclitaxel (5 mg/kg, once every 3 days), or KCP10043F (5, 15, or 30 mg/kg, every day for 5 days) for 27 days. (**B**) Tumor volume (mm^3^) and (**C**) body weight (g) were measured throughout the experimental period. (**D**) The tumors were separated and (**E**) weighed after the mice were sacrificed. (**F**) Immunohistochemical staining of p-STAT3 in the tumor sections was performed. Tumor tissues were homogenized at 27 days after KCP10043F treatment, Western blot analysis showed the inhibition of p-STAT3 (Y705), survivin, and Mcl-1 in whole cell extracts from mice tissue. β-actin was used to verify equal protein loading. (**G**) Apoptosis induction was examined by TUNEL assay using tumor sections. After the administration of KCP10043F for 27 days, tumor tissues were homogenized and lysed to prepare the whole proteins for Western blotting analysis detecting the protein expression of Bcl-2 and PARP. β-actin was used as an internal control.

We then examined the therapeutic potential of KCP10043F for reducing the growth of subcutaneously implanted A549 cells in nude mice. The experimental protocol is shown in Figure 7A. Tumor diameters were measured three times per week, and the animals were sacrificed after 5 weeks. To evaluate the inhibitory effect of KCP10043F in an animal model, we constructed the xenograft model using the A549 cell line and orally administered three different concentrations of KCP10043F for 4 weeks. We found that the tumor volume was decreased in the KCP10043F (5, 15, or 30 mg/kg, p.o., 5 times daily for 4 weeks) and paclitaxel (5 mg/kg/day, i.p., once every 3 days for 4 weeks) treatment groups, compared with the vehicle-treated control group. In particular, the tumor volume in the KCP10043F-treated group (15 or 30 mg/kg) was significantly lower than that in the vehicle-treated control group on day 27 after treatment without the loss of body weight (Figure 7B,C). In addition, compared with the tumor weight in the vehicle-treated control group (0.954 ± 0.294 g), the tumor weight in the KCP10043F-treated groups was reduced (5 mg/kg KCP10043F-treated group: 0.815 ± 0.092 g; 15 mg/kg KCP10043F-treated group: 0.689 ± 0.144 g; 30 mg/kg KCP10043F-treated group: 0.629 ± 0.151 g, *p* < 0.05) (Figure 7D,E). We also investigated whether KCP10043F can modulate the induction of apoptosis and expression of various STAT3-regulated oncogenic gene products in tumor tissues. Treatment with KCP10043F reduced p-STAT3 levels (immunohistochemical staining) and STAT3-regulated proteins such as survivin and Mcl-1 (Western blotting) (Figure 7F). In addition, consistent with the cell-based results of A549 and NCI-H358 cells, TUNEL assays and Western blots of Bcl-2 and PARP studies revealed that KCP10043F enhanced the induction of apoptosis in the tumor tissues of the xenograft model (Figure 7G).

## 4. Discussion

The characteristics of cancer include selective growth, proliferative advantage, invasion, and metastasis [35]. Recently, increasing evidence has suggested that cancer cells uncommonly express the T-type Ca^2+^ channel and the inhibition of this channel may induce apoptosis and reduce cancer cell proliferation [36,37]. We have also found that 3,4-dihydroquinazoline derivatives could suppress the lung, ovarian, and pancreatic cancer cells [20,21,38,39,40,41]. In particular, our previous study showed that KCP10043F (10 µM for 48 h) could exhibit T-type calcium channel blocking activity, induce cell cycle arrest, and cause synergistic apoptotic cell death in combination with etoposide [22]. However, the molecular mechanisms involved in KCP10043F-induced apoptosis remain unclear. As a major mechanism of chemotherapy inducing cell death is commonly known to induce apoptosis, regulation of the apoptotic pathway is the focus of various preclinical drug discovery studies [42]. In the present study, we investigated whether KCP10043F (20 µM for 24 h) can be used as a chemotherapeutic agent for lung cancer treatment by unraveling the mechanism of KCP10043F-induced apoptosis in A549 and NCI-H358 NSCLC cells.

A MTT cytotoxicity assay showed that the effects of KCP10043F on A549 and NCI-H358 cells (IC_50_; 8.23 ± 0.34 μM and 9.23 ± 0.53 µM, respectively) were similar. In the present study, our results revealed that KCP10043F induced apoptosis, displaying the externalization of PS and DNA fragmentation in both lung cancer cell lines. Apoptosis occurs through specific cellular progression, and a major process of this system is a proteolytic system involving caspases, a highly conserved family of cysteine proteases with specific substrates. In general, the initiator caspases such as caspase-8, caspase-9, and caspase-10, activate the caspase cascade and effector caspases such as caspase-3, caspase-6, and caspase-7, cleave several vital substrates such as PARP and lamin A/C, leading to apoptosis [43]. In this study, we found that KCP10043F activated caspase-8, caspase-9, and caspase-3, resulting in the PARP cleavage. Moreover, z-VAD-fmk (broad-caspase inhibitor) was found to significantly inhibit KCP10043F-induced apoptotic cell death. It is well known that apoptosis mediated by caspases is induced via the intrinsic pathway that occurs through the mitochondria or the extrinsic pathway initiated by the death receptor [44]. The disruption of *ΔΨm* triggers the intrinsic pathway, and subsequent release of pro-apoptotic factors such as cytochrome *c* initiates the formation of the apoptosome complex and caspase-3 activation [45]. The Bcl-2 family is divided into three groups based on their primary function (1) anti-apoptotic proteins (Bcl-xL, Bcl-2, and Mcl-1), (2) pro-apoptotic pore-formers (Bax and Bak) and (3) pro-apoptotic BH3-only proteins (Bid, Bad, Bim, Noxa, and Puma). Anti-apoptotic proteins inhibit the oligomerization of pro-apoptotic proteins, leading to permeabilized mitochondria. However, BH3-only proteins can directly (Bid and Bim) or indirectly (Bad) activate Bax and Bak [46]. In the present study, we found that KCP10043F reduced the expression levels of Bcl-2, Bcl-xL, and the pro-form of Bid and enhanced the levels of Bax, indicating that modifications in Bcl-2 family protein expression contributed to the change in *ΔΨm* and resulted in the release of cytochrome *c*. Bid, a pro-apoptotic protein involved in Bcl-2 family member, can be truncated by caspase-8. The carboxyl-terminal fragment of Bid then translocates to the mitochondria resulting in the release of cytochrome *c* [47]. In this study, a reduction of expression levels in the pro-form of Bid indicated that it was cleaved during KCP10043F-induced apoptosis. Although the loss of *ΔΨm* following KCP10043F treatment seems to suggest that caspase-8 contributes to activate caspase-9 via cytochrome *c*, further studies are necessary to elucidate the precise mechanism of the death receptor-triggered apoptotic pathway.

STAT3 is one of the transcriptional factors that play an important role in cancer growth and proliferation [48]. Constitutive activation of STAT3 has been observed in various tumors including lung cancer, breast cancer, prostate cancer, brain tumor, head and neck squamous cell carcinoma, and colon cancer [49,50,51,52]. Therefore, the inactivation of STAT3 has been considered as a promising cancer therapeutic strategy. We found that KCP10043F inhibited both constitutive and IL-6-induced STAT3 (Y705) activation, and it transcriptionally regulated the expression of proteins such as survivin and Mcl-1. This inhibition was also confirmed in EGFR mutant H1975 and HCC827 cells (Figure S3). Moreover, it was found that KCP10043F inhibited nuclear translocation of p-STAT3 and STAT3 in A549 cells (Figure S4). 

IL-6 is a pro-inflammatory cytokine produced in tumor cells and activates the STAT3 pathway through binding to the IL-6 receptor [53]. We found that KCP10043F reduced IL-6 production and IL-6-induced STAT3 phosphorylation. Furthermore, the overexpression of exogenous STAT3 markedly reduced KCP10043F-induced apoptotic cell death. Here, the inhibition of KCP10043F on constitutive STAT3 activation was also investigated by in silico docking analysis. It is well known that STAT proteins are recruited to the receptor through binding to the SH2 domain, which results in the phosphorylation of tyrosine 705 on a carboxy-terminal either directly by the receptor or by a receptor-associated JAK kinase for its activation and dimerization. Then, dimerized STAT3 translocates to the nucleus and binds to specific promoter sequences in target genes [54]. Docking analysis of the interaction of KCP10043F with STAT3 revealed that the binding of KCP10043F to the SH2 domain prevents STAT3 phosphorylation and inhibits STAT3 activation.

A xenograft model was established using A594 cells to validate the in vitro results of KCP10043F in an animal model. Treatment with KCP10043F significantly suppressed tumor growth in the xenograft lung cancer model through apoptosis induction without body weight loss. In agreement with in vitro results, KCP10043F also significantly downregulated the phosphorylation of STAT3 and the expression of various STAT3-regulated genes in mouse tissues.

Our results revealed that KCP10043 suppressed the expression of STAT3-mediated genes involved in cell cycle progression (cyclin D_1_) and anti-apoptotic genes (Bcl-2, Bcl-xL, survivin, Mcl-1), inhibited cell proliferation, caused the accumulation of cells in the G_1_ phase and induced substantial apoptosis in A549 and NCI-H358 cells. Taken together, our study demonstrated the therapeutic potential of KCP10043F for treating human NSCLC. 

## Supplementary Materials

The following are available online at https://www.mdpi.com/2077-0383/9/3/704/s1, Figure S1: Induction of apoptosis by KCP10043F in non-small lung cancer cells, Figure S2: Effect of KCP10043F on STAT3-related proteins in non-small lung cancer cells, Figure S3: Effects of KCP10043F on IL-6-induced STAT3 phosphorylation in EGFR mutant H1975 and HCC827 cells, Figure S4: Effects of KCP10043F on nuclear translocation of p-STAT3 and STAT3 in A549 cells.
