# Supplementary material for: KCP10043F Represses the Proliferation of Human Non-Small Cell Lung Cancer Cells by Caspase-Mediated Apoptosis via STAT3 Inactivation
Source: J Clin Med. 2020 Mar 5;9(3):704. doi: 10.3390/jcm9030704 (PMC7141374; doi:10.3390/jcm9030704)
Supplement: Supplementary file 1 [file jcm-09-00704-s001.pdf]

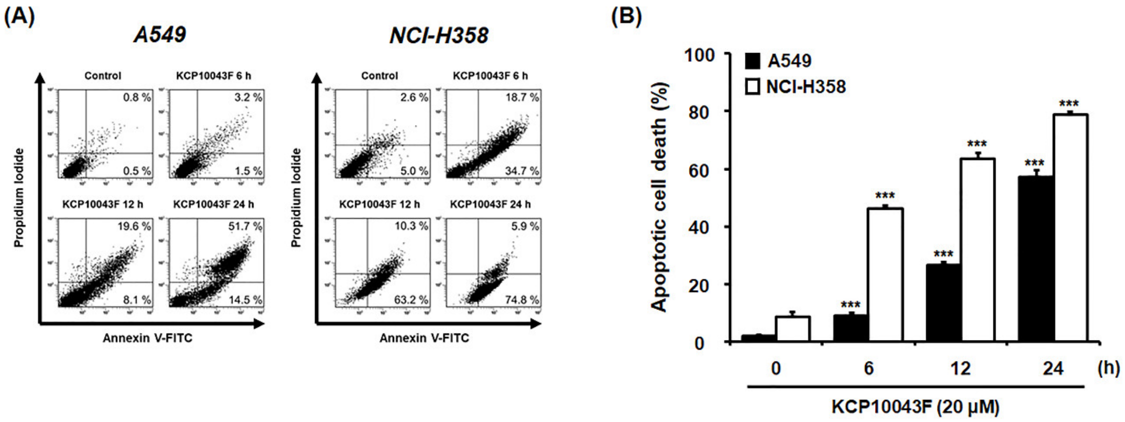

Figure S1. Induction of apoptosis by KCP10043F in non-small lung cancer cells. (**A**) A549 and NCI-H358 cells were treated with 20 μM KCP10043F for indicated times (6, 12, or 24 h) and co-stained with PI and FITC-conjugated annexin V for detecting apoptosis by flow cytometry. (**B**) The portion of early apoptosis ($Annexin^+/PI^-$) cells and late apoptosis ($Annexin^+/PI^+$) cells in the graph is determined as apoptotic cell death rate. Data represent the mean ± SD of the results from three independent experiments. *** $p < 0.001$ vs. untreated control group.

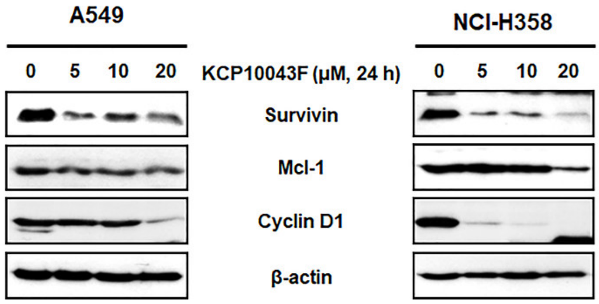

Figure S2. Effect of KCP10043F on STAT3-related proteins in non-small lung cancer cells. A549 and NCI-H358 cells were treated with KCP10043F (5, 10, or 20 μM) for 24 h. Total cell lysates were prepared and fractionated by SDS-PAGE, transferred onto PVDF membranes, and examined by using primary antibodies specifically detecting cyclin D_1, Mcl-1, and survivin. β-actin was used as an internal control. The experiment was repeated three times, and similar results were obtained.

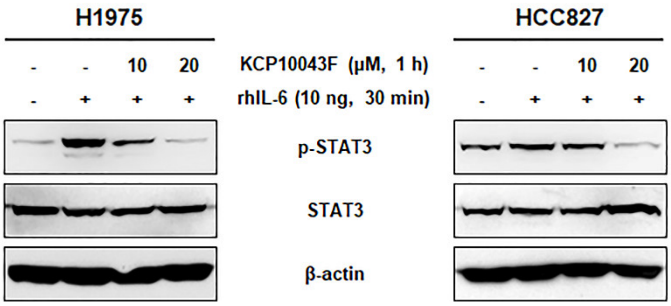

Figure S3. Effects of KCP10043F on IL-6-induced STAT3 phosphorylation in EGFR mutant H1975 and HCC827 cells. Cells were treated with 20 μM KCP10043F for 30 min and then treated with 10 ng human recombinant IL-6 for an additional 30 min. Total cellular proteins were resolved by SDS-PAGE, and p-STAT3 and STAT3 were detected using specific antibodies. β-actin was used as an internal control.

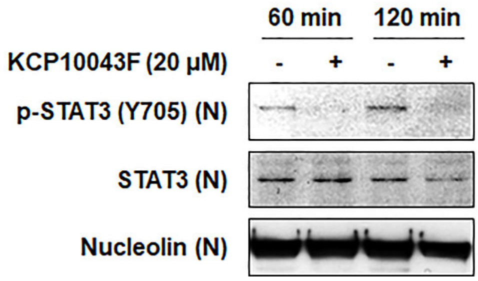

Figure S4. Effects of KCP10043F on nuclear translocation of p-STAT3 and STAT3 in A549 cells. Cells were treated with 20 μM KCP10043F for indicated times (60 or 120 min). Total nuclear proteins were resolved by SDS-PAGE, and p-STAT3 and STAT3 were detected using specific antibodies. Nucleolin was used as an internal control.
